# The Experience of Nursing Instructors and Students on Professional Competency of Nursing Academic Staff: A Qualitative Study

**DOI:** 10.5539/gjhs.v6n4p128

**Published:** 2014-04-13

**Authors:** Hedayat Jafari, Eesa Mohammadi, Fazollah Ahmadi, Anoshiravan Kazemnejad, Seyed Afshin Shorofi

**Affiliations:** 1 Department of Nursing, Faculty of Medical Sciences, Tarbiat Modares University, Tehran, Iran; 2 Department of Biostatistics, Faculty of Medical Sciences, Tarbiat Modares University, Tehran, Iran; 3 Department of Nursing, Faculty of Medical Sciences, Mazandaran University of Medical Sciences, Sari, Iran; 4 Flinders University, Adelaide, Australia

**Keywords:** professional competency, nursing academic staff, nursing students, qualitative study

## Abstract

**Background:**

One of the most important purposes of health-oriented educational centers is to train competent nurses. This study was intended to discover the meaning of “competency of nursing academic staff” among nursing students and instructors.

**Methodology & Methods:**

A qualitative study using the content analysis approach was conducted. The data was collected through in-depth interviews consisting of 30 individuals and a focus group.

**Results:**

Data analysis resulted in the extraction of 15 categories and 6 themes. The themes included ‘providing effective education’, ‘increasing research capability of oneself and that of the students’, ‘promoting managerial competency’, ‘being a cultural and behavioral role model’, ‘motivating and nurturing the students’, and ‘boosting teaching values and disposition’.

**Conclusion:**

This study resulted in discovering the real meaning of professional competency among the nursing faculty staff. The findings could be utilized in designing assessment tools for professional competency of nursing academic staff in nursing schools and other medical fields.

## 1. Introduction

Competency is a controversial issue and a complicated concept in education and health that is considered highly important in various fields of nursing. Many studies are conducted in this field and many definitions are provided, however, ambiguities still exist in understanding this concept (O’Neale & Kurtz, 2001; Ravari, Bazargan, Vanaki, & Mirzaei, 2012).

Alexander and Runciman (2003) have proposed a practical definition of professional competency in nursing. They believe that competency in nursing is a combination of specific knowledge; behaviors and skills as they relate to cognitive, affective and psychomotor domains of practical nursing. Different studies have suggested various definitions for professional competency which show lack of agreement on this concept. The reason behind all these differences can be due to diverse attitudes, patterns, experiences and areas in viewing this novel phenomenon that the origin of all definitions is by gaining competency.

According to Lindop (1987), nursing instructors ought to be aware of the students’ attitude towards educational development. Therefore, their experiences and feelings have to be taken into account. Harris (1998) believed that effective training depends on educational outputs and requires scientific qualifications, skills, behaviors, educational styles, professional development, and research. In this respect, the role of the competent instructor is highly significant.

The studies have revealed that there is a deep gap between classical nursing training process and clinical performance so that the existing instructions do not give the student the necessary ability to achieve clinical skills (Hadizadeh, Firoozi, & Shamaeyan, 2005). In fact, the importance of professional competencies of nursing educatorshas been neglected by the profession. Regarding the significance of professional competencies of nursing educators, this study was conducted to discover the meaning of “competency of nursing academic staff” among nursing students and instructors. Professional competency is believed to be very important for nursing academic staff. So, this study aimed at investigating this issue considering the learner’s and the instructor’s perception and experiences in their particular organizational, cultural and social background.

## 2. Methodology & Methods

### 2.1 Design

The aim of this study was to investigate the meaning of professional competency among nursing academic staff and students. Thus, content quality analysis was used to discover and explain human feelings and meanings hidden in daily experiences (Polit, 2010).

### 2.2 Participants

The study participants included nursing faculty staff who have been evaluated at least once by their students and also students who have experienced evaluating their instructors at Mazandaran University of Medical Sciences. The interview location was determined by the participants’ which was mostly the instructors’ workplace or the students’ education place. Purposeful sampling was done between July 2012 and June 2013 until data saturation (Table 1).

**Table 1:** Participants’ characteristics

Characteristics	Participants		

	Students	Focus group	Instructors
Educational level and scientific rank(N)*	Bachelor(10)	BSc. Student(6)	Assistant Professor(4)
	MSc(4)		Instructor(4)
	PhD(2)		
Age(years)	20-50	21-26	42-55
Gender(N)	Male(8)	Male(3)	Male(4)
	Female(8)	Female(3)	Female(4)
Experience(years)	-	-	13-30

* Number

### 2.3 Data Gathering and Analysis

In this study 24 in-depth interviews including open-ended questions and six complementary interviews for clarification were conducted. One focus group interview was also carried out involving six individuals of the participating students. Some of the main questions posed for starting individual interviews were as follows:

- As a faculty staff, what duties do you have and how are you going to perform them?
- In order to perform these duties, what abilities and qualifications do you create in yourself?
- If possible, would you please explain more about these abilities and qualifications with some objective examples to understand you better?
- As a student, which aspects and features are more important to you when evaluating your instructor?
- What positive characteristics do you look for in your instructor?

Then based on the answers, the interviews deepen by posing questions such as” would you please explain more? Or “what do you mean by that?”

Each interview took 40-60 minutes and the complementary interviews varied between 5 and 15 minutes (Sandelowski, 2000). The focus group interview consisting of 6 nursing students started asking ‘what experiences do you have from your competent instructor in classroom or clinical training?’ Then the participants were encouraged to express their perceptions and experiences while being guided by the researcher.

Data analysis was deductive, comparative and a continuous process, simultaneously performed with data collection. The process of data analysis was as follows: preparing data, making decision about unit analyses, categorizing codes, encoding a sample of the text, encoding all text, evaluating encoding stability, concluding from the encoded data, and findingsreported. All tape-recorded interviews were transcribed into text as soon as possible after each interview. The transcribed interviews were reviewedseveral times to gain an understanding of the interviews. The meaning unitswere then underlined andsubsequently reviewed several times, followed by allocating a code for meaning unit. The codes were subsequently categorized based on their commonmeanings and were condensed as much as possible. Finally, data constructed major categories which were more general, leading to form the themes. Data analysis process was repeated after the inclusion of subsequent interviews and the categories were then modified accordingly.

### 2.4 Trustworthiness

To increase the validity of the data, the participants were given the transcribed interviews and the primary codes to give their ideas. Afterwards, essential changes based on the cases’ suggestions were applied. Two experts in the field of qualitative studies have monitored the research process. Sampling was carried out with maximum variance enhancing data confirm ability, dependability, and transferability (Sandelowski, 2000).

### 2.5 Ethical Considerations

The ethical consideration was approved by ethical committee of the Tarbiat Modares University of Medical Sciences. The oral consent was taken from the participants before the interviews. They were assured that their statements would remain confidential.

## 3. Results

Data analysis resulted in 242 primary codes, 15 categories and 6 themes. The themes encompass: “providing effective education, increasing the research capability of oneself and that of the students, enhancing managerial competency, being a cultural and behavioral role model, motivating and nurturing the students, and boosting mastership values and disposition”.

### 3.1 Providing Effective Education

This theme shows the focus of instructors to important criteria in order to promote education. Of such criteria are paying attention to teaching methods and using educational technology to help them make lesson-based material understood. As one teacher said:

“I use educational technology and appropriate teaching methods”. (P.1, 13 years experience)

Making the class fresh and attractive is of the significant qualities that the majority of the participants found highly important in education.

“I manage the class in a way that the students don’t feel tired or bored.” (P.3, 19 years experience)

One of the issues done by the participants to provide effective education was having plans in instructing the students.

“I plan for my classes.” (P.2, instructor, 25 years experience)

“The instructors who had plans during the course were more successful.” (P.19, MSc student, semester 1)

Nursing students and instructors believe in the teachers’ up-to-date knowledge as an important quality. one of the nursing instructors stated:

“I keep myself up-to-date.” (P.30, 19 years experience)

The participants consider experience highly influential in transferring concepts. One of the experienced instructors said:

“I have certainly gained more experience every year.” (P.2, 50 years old, 25 years experience)

One of the most effective ways to improve the quality of clinical instruction is to supervise and accompany the students in the clinical environments:

“*He answered the students’ questions in clinical centers.” (P.9, student in semester 8)*

Having a rational criterion for evaluating the students is completely appreciated by the instructors:

“In evaluation, I take all of the student’s activities into account.” (P.2, teacher, 25 years experience)

The aforementioned points raised by nursing instructors and students indicated a high value of efficiency and usefulness of nursing theoretical and clinical instructions, which enhance scientific competency and helps in gaining specialized skills and transferring them to the students.

### 3.2 Increasing One’s Research Capability and That of the Students

This theme implies that instructors pay special attention to particular criteria to promote their researches. For instance they perform specialized nursing researches to enhance their knowledge:

“I do nursing studies related to my field.”(P.1, 13 years experience)

The students also had similar ideas:

“To do my research projects, I write proposals along with my instructor.” (P.19, MSc student, semester1)

Moreover, the participants focus on publishing their studies.

“I published many papers and authored a book too.”(P.8, 26 years experience)

Acquiring and enhancing research capability has been emphasized by both nursing instructors and students. One of the instructors stated:

“I am really interested in research.”(P.6, 22years experience)

“I try to be proficient at research methods.”(P.7, 18 years experience)

The experiences and feelings expressed by instructors and students indicate that the instructors’ efforts to perform valuable studies, publishing them, boosting their research capability and transferring them to students are amongst the main factors increasing the instructors’ research potential and that of the students.

### 3.3 Enhancing Managerial Competency

Among the features that the instructors try to create and strengthen in themselves is to accept responsibility and to fully carry out their assigned duties:

‘I try to acquire management skills.”(P.10, 13 years experience)

Our study population considers participating in various educational meetings as one of the duties of the instructors and one of the criteria based on which a competent instructor is judged:

“*As the head of department I take part in educational meetings.”(P.2, 25 years experience)*

Recognizing and observing nursing regulations and standards are also emphasized by both instructors and students:

“I try to follow the professional standards in my major.”(P .1, teacher, 13 years experience)

Having social and political relationships with influential university and country authorities is also considered very important. Instructors see this as one of the ways to promote nursing profession:

“In nursing the communication between the patient, colleagues, student and related authorities is of great importance.”(P.4, teacher, 24 years experience)

“I communicate with executive officials to get social support for my profession.”(P.1, 13 years experience)”

Such points define enhancing managerial competency as accepting managerial responsibilities, trying to attend specialized meetings, recognizing nursing principles and standards, adhering to professional guidelines and making efforts to facilitate and improve communications.

### 3.4 Being a Cultural and Behavioral Role Model

This theme suggests that instructors pay special attention to significant criteria in order to promote their cultural and behavioral level. Such criteria include attending cultural meetings and boosting their cultural aspect of personality.

“I try to strengthen myself in cultural aspects.”(P.6, 22years experience)

The majority of the nursing instructors try to transfer their cultural and behavioral experiences to their students. Instructor stated:

“I usually start my class with a very good and proper cultural example.” (P.3, 19 years experience)

“I’ve learned many cultural points from my instructor.” (focus group)

By being a role model for the students through conducting acceptable behavior and actions, nursing instructors try to develop their characteristics in many aspects:

“My moral behavior will teach the students many points.” (P.4, 24 years experience)

The instructor’s attention and practical efforts on attending cultural meetings, having logical religious beliefs, moral behavior, transferring appropriate behaviors to the students, and being a decent role model will help in shaping the student’s character.

### 3.5 Motivating and Nurturing the Students

Theme indicates the teacher’s capability in motivating and supporting nursing students comprehensively.

This One of the features paid attention by the participants was to motivate the students:

“I always encourage my students and motivate them.” (P.7, 18 years experience)

One student also said:

“It’s very pleasant to work with instructors who try to increase the student’s self-confidence “(P.9, semester 8)

The students also believe that they should receive consultation from their instructors. This in fact could help them in developing critical thinking:

“I referred to my teacher and received supportive consultation whenever needed.”(P.10, semester 8)

According to the participants this theme includes mutual respect between the teacher and student, being logical about educational affairs, having respect for the students, being tolerant when communicating with students, and motivating and supporting the students.

### 3.6 Boosting Mastership Values and Disposition

Last theme according to participants’ perception was improving mastership values and disposition.

Moral values are considered very important by instructors and students:

“I expect my instructor to be fair in all aspects.” (P.1, semester 8)

“I have a good behavior with my students; I am very much focused on moral values.”(P.3, 19 years experience)”

Another important feature that highly benefits the students is to feel responsible towards them. One of the instructors said:

“I respect my students’ feelings and try to support them.” (P.7, 44 years old, 18 years experience)

The students expect their instructors to understand and guide them:

“My instructor understands me well and guides me.”(P.10, semester 8)

Analyzing and finding the reasons for student’s failure was also considered significant. One of the students said:

“One of my instructors analyzed and discovered my source of failure.” (P.15, semester 4)

These examples clearly show that instructors’ moral values and characteristics can do much in enhancing educational process. In sum, the study demonstrated that the majority of nurse instructors and students had almost the same perception and experience concerning professional competency of nurse instructors.

## 4. Discussion

This study revealed that the experiences of nurse instructors and students about professional competency involve special domains and characteristics including: “providing effective education, increasing the research capability of oneself and that of the students, enhancing managerial competency, being a cultural and behavioral role model, motivating and nurturing the students, and boosting mastership values and disposition”.

The feelings and perceptions pointed out are the reflection of two classes of experiences, perceptions and feelings of the instructors and those of the students that in most cases similar ideas were observed.

In a study on the viewpoints of students about a competent lecturer, having teaching experience, having general and specialized knowledge, and being up-to-date were considered highly significant (Mazloomi, Rahaie, & Ehrampoosh, 2010). Also, a study conducted by Parsayekta, Ahmadi, and Tabari (2005) about the factors influencing acquiring clinical competency from the nurses’ point of view signified the role of experience as the most influential factor. Arbon (2004) stated experience as the unique and distinct events that an individual acquires during a complicated process called meaning making. We observed our findings consistent with Parsayekta et al. (2005) and Mazloomi et al. (2010) regarding providing effective education, having experience and being up-to-date. In mentioned studies, the importance of concepts in basic courses, presenting material in a logical manner and teaching in a simple language that are related to the same theme have not been dealt with.

In the present study, nursing lecturers and students have pointed out knowledge, high level of capability in teaching, being up-to-date, and attending the class with sufficient information as the main characteristics of a competent nursing instructor. These points were also observed by other studies (Sanagoo, Joybari, & Nikbakht, 2002; Rashadmanesh, Zarezadeh, & Hajir, 2007). In a study by Amini and Momennasab (2003), the instructor’s knowledge was found the most important characteristic of a competent nursing instructor and this is consistent with “providing effective education” found in this study. However, that study did not include effective clinical training.

This study’s participants believed in good rhetorical and teaching skills, the instructor’s proficiency, and his interest in teaching as the most important factors affecting their perception about a competent nursing instructor. Mandira, Farouk and Abdulbari (1996) also found knowledge and proficiency as the main qualities of an ideal instructor.

Bagheri-Nesami, Rafiei, Parvizi and Esmaeili (2008) in their research described theoretical knowledge as a significant factor for promoting clinical instruction competency and introduced it as a catalyst. In their research, in addition to theoretical knowledge, experience was observed as the most influential factor in acquiring clinical competency which is created by practice and repetition, which is in line with our findings.

Their study showed that feeling responsible for the students and supporting them could play an important role in the communication between instructors and students. Furthermore, being available, having a close relationship with the student, and wining the student’s trust reveal the teacher’s competency under the theme “boosting mastership values and disposition”. Siamian et al. (2012) and Gashmard et al. (2011) also found similar results and realized that students could establish a close relationship with the instructor who benefits from problem solving skill and this is consistent with our study too.

Gaining social and state support and drawing the attention of political and social authorities’ to the nursing profession in both educational and clinical fields can improve the competency of nursing faculty staff in managerial dimension. The studies conducted by other researchers also showed that nurses need social support (Laschinger, Finegan, Shamian, & Casier, 2000; Brown & Carolyn, 2002; Adib, Salsali, & Ahmadi, 2004). It is believed that nurses do not receive enough social and political supports. We believe self-confidence is of the factors influencing the level of using learning opportunities. In this research, in the “motivating and nurturing the student” theme motivating and enhancing the students’ self-confidence are considered very important. Other studies also found similar results (Eraut et al., 2004; Lee-Hsieh, Kao, & Tseng, 2003; Parsayekta et al., 2005).

The concept of competency in nursing is manifested through acquiring and developing educational, managerial, cultural, moral, research, and individual skills and then evaluated based on these factors. Exploring this concept through qualitative studies could highly benefit nursing fields (both educational and clinical).

The present qualitative study only provides the perspectives of a group of nursing students within the Iranian culture and context. Therefore, the transferability of findings should be considered with caution and critiqued and compared with those of similar studies conducted in other contexts. Further studies in different cultures and contexts should be conducted to help us recognize various aspects of professional competency of nursing academic staff.

## 5. Conclusion

This study resulted in discovering the real meaning of professional competency among the nursing faculty staff. As stated competent instructors are those who make efforts to provide effective education, increase the research capability of oneself and that of the students, enhance managerial competency, are cultural and behavioral role models, motivate and nurture the students, and boost mastership values and disposition while these efforts are simultaneously perceived by the students. This definition is different from the definitions presented in other qualitative studies.

The present research is the result of a survey based on real experiences and perceptions of the main groups involved in nursing field, therefore, the findings could be utilized in designing assessment tools for professional competency of nursing academic staff in nursing schools and other medical fields.
